# Alleged malpractice in orthopaedics. Analysis of a series of medmal insurance claims

**DOI:** 10.1186/s10195-018-0500-4

**Published:** 2018-07-27

**Authors:** M. B. Casali, A. Blandino, S. Del Sordo, G. Vignali, S. Novello, G. Travaini, M. Berlusconi, U. Genovese

**Affiliations:** 1 Sezione di Medicina Legale e delle Assicurazioni—Dipartimento di Scienze Biomediche per la Salute, Università degli Studi di Milano, Via Luigi Mangiagalli 37, 20133 Milan, Italy; 2 Humanitas Research Hospital, Trauma, Milan, AO Italy

**Keywords:** Malpractice, Orthopedics, Insurance claim review, Liability, Forensic medicine

## Abstract

**Background:**

Medical malpractice is an important topic worldwide, and orthopedics is a clinical branch that is considered to be at a high risk for claims. The analysis of a series of medmal insurance claims allows forensic pathologists, clinicians, and insurance companies to probe the risk of a specific clinical branch for medical malpractice claims and highlights areas where care may be improved. We investigated the main features of a major Italian insurance broker’s archive in order to identify recurrent pitfalls in this field.

**Materials and methods:**

A retrospective study was carried out on orthopedics claims. The archive covered claims from 2002 to 2013 that targeted 1980 orthopedists.

**Results:**

635 claims were found and analyzed with a focus on the clinical activity invocked in the claim, the presence of alleged team malpractice, the clinical outcome of the case, and the final forensic decision regarding the claim. 299 orthopedists had at least one malpractice claim made against them during the available period; 146 orthopedists were subject to more than one malpractice claim. Most of the claims regarded perioperative and operative cases, usually originating from civil litigation. The anatomical sites most commonly involved were the hip or knees, and sciatic nerve lesions were the main contributor.

**Conclusions:**

Orthopedics is a medical specialty with a high risk for malpractice claims. In our study, medical malpractice was observed in nearly 50% of the cases—typically in surgery-linked cases resulting in permanent impairment of the patient. Death from orthopedics malpractice seemed to be rare.

**Level of Evidence:**

IV.

## Introduction

Medical malpractice (medmal) is currently an important topic in forensic pathology [1], and the traditional medicolegal approach to this subject is to analyze the various clinical branches separately first in order to simplify the description of a very complex phenomenon, and then to improve and optimize clinical risk management strategies [2].

Orthopedics, a major surgical specialty, is a clinical branch that is considered to be at a higher risk for medical malpractice claims [3, 4].

Analysis of a huge series of medmal insurance claims allows forensic pathologists, clinicians, and insurance companies to ponder the intrinsic risk of a specific clinical branch for medical malpractice claims. Malpractice claims are not the same as defined judicial judgements, as the former relate mainly to patients’ dissatisfaction whereas the latter relate to the ascertainment of the alleged clinical mistakes. Indeed, a malpractice claim is the invariable first step towards a hypothetical medmal blame, while the judicial judgement is its eventual outcome after potentially many years and various chances to resolve the claim extrajudicially. Therefore, the analysis of a series of malpractice claims (versus the analysis of a series of judicial judgements) leads to greater knowledge of the medmal phenomenon and all of its dynamics, knowledge that is also beneficial in the fields of forensic pathology and clinical risk management [5, 6]. Moreover, studies of the pre-trial management of malpractice claims can help to bridge the research gap between works describing the epidemiology of claims (as in the present investigation) and works analyzing final judicial judgements.

The aim of the present study was to analyze claims regarding alleged orthopedic malpractice through the anonymized study of the claims database of one of the largest Italian private insurance brokers, in order to map professional risk and identify recurrent pitfalls.

## Materials and methods

A retrospective study was carried out on the basis of an archival data analysis of one of the largest medmal insurance brokers in Italy. The focus of the study was on all the available claims relating to specialized orthopedists. The available archive covered claims from January 2002 to December 2013, and comprised 793 malpractice claims for a total population of 1980 orthopedists. Each of the selected files typically consisted of hospital and medical records, personnel reports, official plaintiff blames, and medicolegal reports. Just 635 orthopedic claims (80% of 793) were considered eligible for the analysis because of the completeness of the available information.

The analysis focused especially on three major aspects: the characteristics of the orthopedist(s) involved (sex, age, and number of claims received), the characteristics of the implicated patient (gender, age, comorbidities), and features of the alleged event of malpractice. In terms of the features of the event, it was assessed whether the claim originated as a result of civil or penal litigation, the type of hospital in which it took place (a specialized orthopedic or general hospital), the day of the week on which it occurred, the presence of alleged team malpractice versus single-physician malpractice (and possibly the other branches involved), the type of event (surgical versus nonsurgical event, perioperative versus intraoperative event, elective surgery versus post-traumatic surgery), and the anatomical site affected. Other declared points of analysis were the clinical outcome of the case (death versus permanent impairment) and the final forensic decision on the claim (confirmed malpractice versus rejected malpractice). Finally, attention was also focused on the presence of nosocomial infections and pulmonary thromboembolism, two common and crucial topics in orthopedics. Cases that did not have this information were excluded.

Problems concerning consent for the procedure and the final costs connected with the confirmed malpractice were not analyzed in the present study.

Finally, a descriptive-statistic approach was employed to describe all the data obtained, using the chi-square test with a statistical cutoff of *p* < 0.05.

## Results

The population of 1980 orthopedists were 90% male, and the mean age of a physician at the time of the first malpractice claim against them was 49.5 ± 11.4 years. Just 299 orthopedists (15.1% of the total population) in the archive had at least one malpractice claim made against them during the available period, while 146 orthopedists in the archive (7.4% of the total population and 46.3% of the 299 orthopedists that had a claim made against them) had been the focus of more than one malpractice claim. Approximately 10% of the 299 orthopedists who received a medmal claim against them had received more than three alleged malpractice claims against them during the available period.

**Fig. 1:**
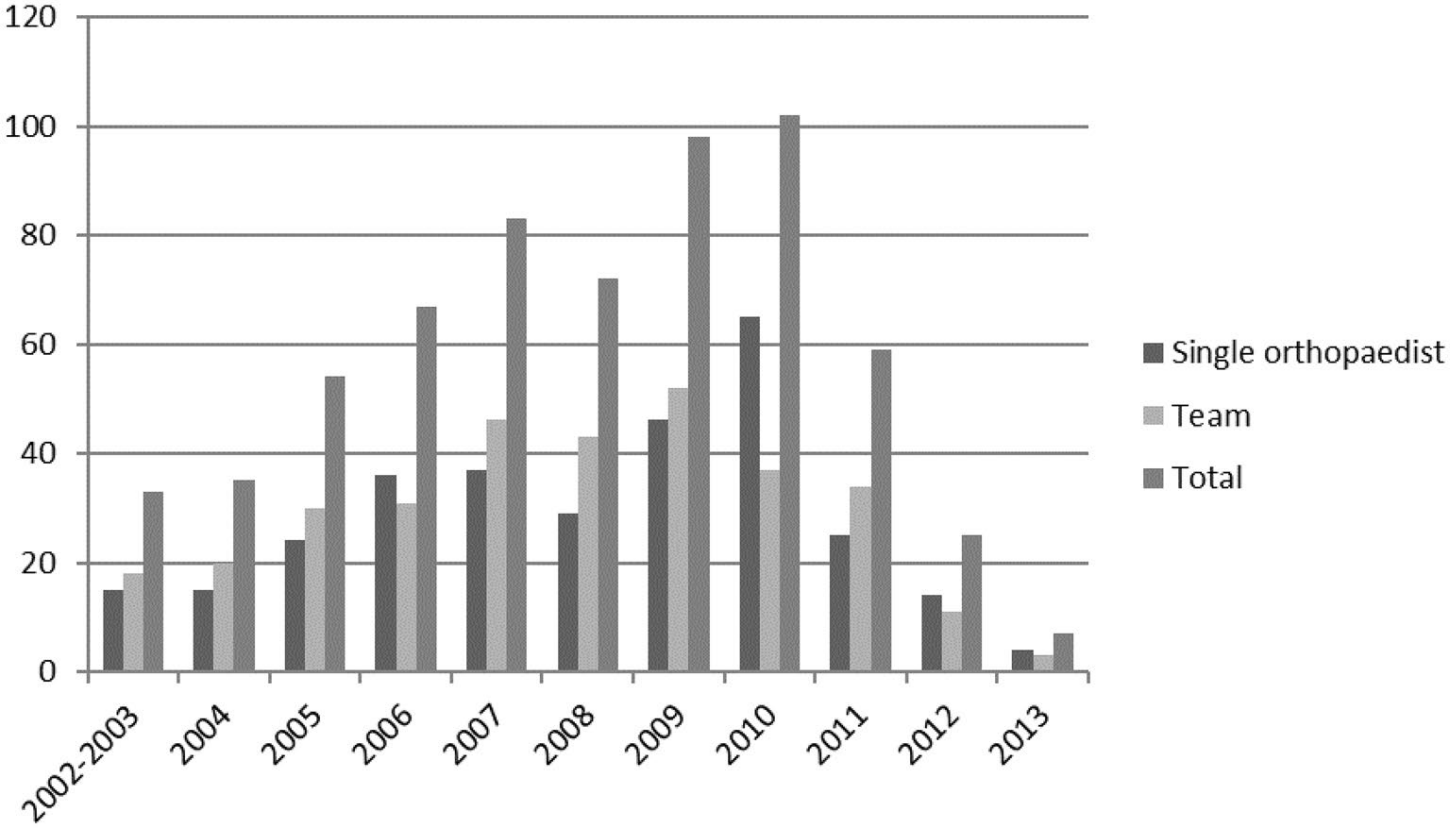
Historical trend in alleged orthopedic malpractice claims

In the five-year interval from 2008 to 2012, the estimated cumulative risk of an orthopedist in the archive receiving at least one malpractice claim against them was 19.3%. During the same period, the mean annual risk that an orthopedist in the archive would be the subject of a malpractice claim was 6%. Figure 1 depicts the historical trend in orthopedic alleged malpractice claims graphically.

Approximately 90% of the claims had been opened as civil litigation. Conversely, all of the claims opened as penal litigation had the patient’s death as the clinical outcome.

The gender distribution in the group of alleged malpractice victims was close to 1:1 (male victims comprised 44%), and the mean age of the victim at the time of alleged malpractice was 53.3 ± 16.8 years. About 8% of the alleged malpractice victims were younger than 30 years. Heart (11%), endocrine (8%), and neuropsychiatric (7%) diseases were the most common comorbidities of the victims of alleged malpractice.

More than 95% of the claims concerned hospital-linked malpractice cases, with general hospitals accounting for 89% and specialized orthopedic hospitals accounting for only 11% (*p* < 0.001). The orthopedists in the archive were equally distributed in terms of primary workplace between general and orthopedic hospitals. Weekend hospital procedures were the target in 12% of all the claims analyzed.

In 485 cases, there was an original allegation of multidisciplinary team malpractice (76.4% of the entire population). Those team malpractice claims were divided into claims targeting only orthopedic teams (51% of all claims) and claims that were also targeting other clinical branches (25.4% of the total claims), in particular anesthesiology and radiology. Claims focusing only on a single specialized orthopedist totaled 311 (49%), while 161 cases targeted a single specialized orthopedist as well as other physicians. Team malpractice claims typically related to surgical adverse events. Claims targeting single orthopedists were mainly focused on wrong diagnoses in the ER setting as well as surgical adverse events. Figure 1 shows the chronological trend for team versus single-physician malpractice claims.

Most (83%) of the claims concerned perioperative and intraoperative adverse events. Nonsurgical adverse events and purely postoperative adverse events constituted a small fraction of the claims (*p* < 0.001).

Sixteen percent of the intraoperative adverse events concerned isolated vascular or nervous iatrogenic injuries to the lower limb: post-traumatic surgery accounted for 25% of the claims and elective orthopedic surgery for the other 75% (*p* < 0.001). Elective prosthetic surgery accounted for 27% of the latter claims, with hip or knee replacement procedures especially prominent (60% and 31% respectively, *p* < 0.001). Shoulder replacement surgery accounted for only 5% of all the prosthetic claims. Claims concerning post-traumatic surgery related mainly to fracture-site nonunions or malunions.

**Fig. 2:**
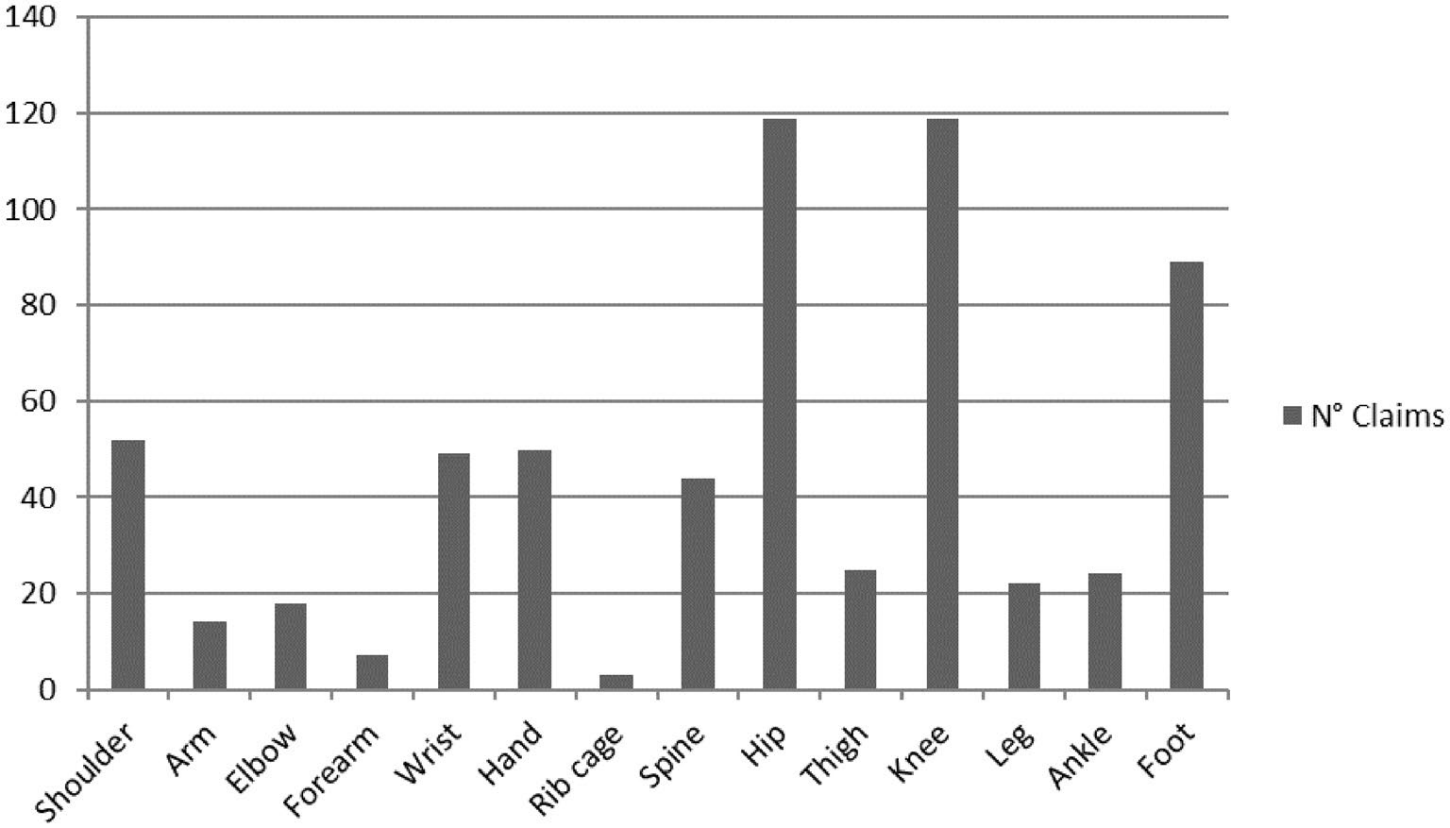
Number of claims per anatomical site

Hips or knees were the surgical sites of interest in nearly 40% of all surgery-linked claims (this value rose to 47% when femur and leg surgeries were included), while shoulders + arms, elbows + forearms, wrists + hands, spine, and ankles + feet accounted for 8 + 2% (*p* < 0.001), 3 + 1% (*p* < 0.001), 7 + 8% (*p* < 0.001), 7% (*p* < 0.001), and 3 + 14% (*p* < 0.001), respectively. About 5.5% of all the analyzed claims related to unsuccessful surgery for hallux valgus (Fig. 2).

Diagnostic mistakes were the main focus in 12% of the claims, with missing diagnoses of lower limb fractures accounting for about 60% of such claims (*p* < 0.015).

Pure postsurgical adverse events (rehabilitation falls, deep vein thromboses, and/or pulmonary thromboembolism) accounted for about 5% of all the claims.

Nosocomial postoperative infections were the source of interest in 14% of all the claims: hip (replacement procedures) or knee (replacement and arthroscopic procedures) surgery was involved in 56% of such claims. About half of the claimed nosocomial infections came from prosthetic surgery. The ratio of post-traumatic to elective surgery in the subgroup of claimed nosocomial infections was 1:3.

A deep venous thrombosis/pulmonary thromboembolism (DVT/PTE) was the main reason for 23 claims (3.6% of all the claims): 18 cases of claimed DVT/PE were postsurgical, with a 1:1 ratio for post-traumatic to elective surgery, and with hip and knee surgery the procedure of interest in 17.

About 9.5% of all claims (60) had the patient’s death as the clinical outcome: 20 patients died of septic shock, 16 because of a pulmonary thromboembolism, 16 due to cardiogenic shock, and 4 because of a major hemorrhage at the surgical site.

**Fig. 3:**
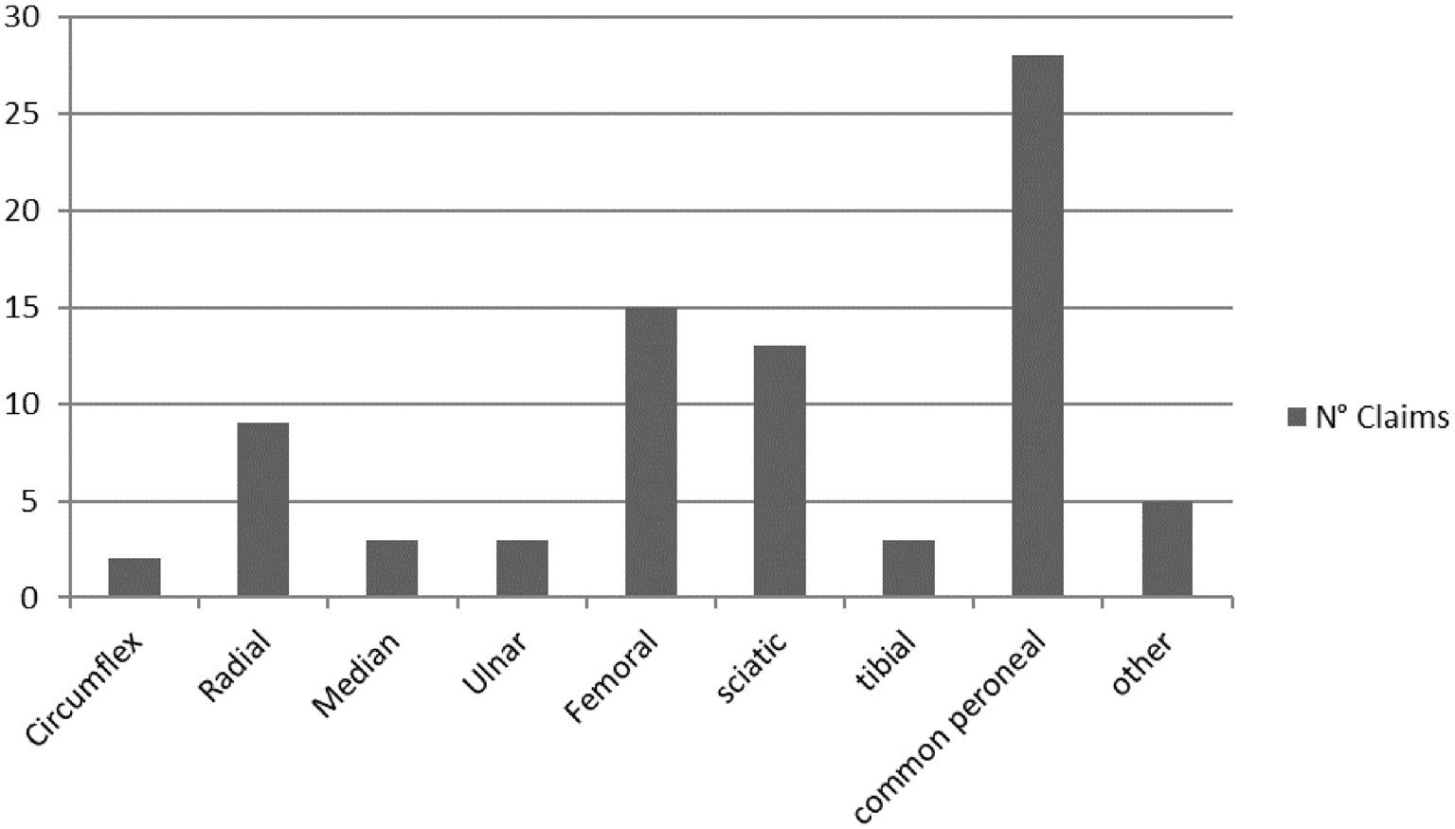
Number of claims concerning nerve injury

Among the claims in which the clinical outcome was permanent impairment (90.5% of all the claims), 81 were intraoperative nerve injury cases, while a sciatic nerve injury (considered to be sciatic + tibial + common peroneal nerve) incurred during the hip replacement procedure accounted for about 50% of such cases (Fig. 3).

Medical malpractice was ascertained in 52% of all claims (330 cases). Medical malpractice was ascertained in just 29% of the 60 claims with patient death as the clinical outcome. Medical malpractice was confirmed (due to wrong prophylaxis and antibiotic therapy) in 60% of the claimed nosocomial infections. Ascertained malpractice was also observed for 22% of the DVT ± PE claims because of a wrong dosage of antithrombotic drugs.

## Discussion

It is well known [7–11] that orthopedics is a clinical branch that is at a high risk for malpractice claims. For example, in the present study, there was found to be a 5-year cumulative risk for claims of close to 20%, and it was noted that 50% of the orthopedists who received claims against them actually had multiple claims lodged against them by the end of their career. Based on a similar insurance archive, the authors have previously estimated the cumulative 10-year risk that a specialized anesthesiologist will be the subject of a malpractice claim at approximately 7%, and the risk that they will be the subject of two or more claims at around 1% [12].

In a wider cross-sectional comparison of different surgical branches, some other Italian authors estimated that the risk of an orthopedic malpractice claim was lower than the corresponding risk for a general or plastic surgeon but higher than the corresponding risk for a gynecologist [7].

Together with the understandable tendency for orthopedic malpractice claims to target staff at general hospitals (as opposed to specialized orthopedic hospitals), these simple statistics highlight the intrinsic risk for claims in a very difficult and challenging surgical branch—one in which physicians must continuously update their techniques. Further, one special feature of orthopedics is its close ties to medical engineering companies that develop artificial implants and prostheses [4], such that orthopedics is commonly believed by patients to be liable for poor artificial device performance too.

As is also evident from the data in the present study, the frequency of orthopedic malpractice claims is on the rise [10, 13, 14]. A more balanced patient-physician relationship (in terms of perceived knowledge) can explain such increase, together with a tendency of some victims of both major or minor perioperative complications to engage in financial speculation [8, 9]. It should be stressed that in the present study problems concerning the consent for a procedure were not considered; however, it should be noted that the previous literature agrees that medical mistakes and negligence in obtaining informed consent can also play important roles [7, 15].

The orthopedic claim data presented here derive mainly from intraoperative adverse events and mainly focus on elective orthopedic surgery. Traumatology accounts for only 25% of the claims, in good agreement with the findings of previous studies [7], possibly because of lower preoperative expectations of acute and hyperacute patients [16]. Indeed, it is believed that the most important reason for malpractice claims is a large negative disparity between the preoperative expectations of the patient and the postoperative results for that patient [7, 17]. In other words, elective orthopedic surgery could be at a relatively high risk of malpractice claims simply because nonacute patients can have greater pretreatment expectations. One of the alleged psychological reasons for malpractice claims following traumatologic surgical procedures is that the patient does not have any feeling of illness or disability preoperatively. Some authors have found that traumatology plays a major role, perhaps as much as elective orthopedic surgery, in generating malpractice claims [13]; for example, in pediatric patients with elbow or other upper limb fractures [14, 18, 19]. Though interesting and useful, we could not specifically analyze child victims of alleged orthopedic malpractice.

Iatrogenic injuries to either nerves or vessels are typical reasons for malpractice claims in orthopedics, together with failed correction of the hallux valgus; unpredicted intraoperative complications and substantial failure of surgical therapy are therefore among the most common starting points for a malpractice claim. Regarding hallux valgus surgeries, up to a third of the patients who underwent curative surgery were found to be dissatisfied with the postoperative result [20]. As already stated in other papers [21, 22], better and longer learning curves for would-be orthopedic surgeons, better preoperative planning, and better preoperative communication with the patient could prevent a significant fraction of the claims. Such changes could positively impact on both technical and nontechnical orthopedic errors [3], limit the bad consequences of the daily activities of the “disruptive orthopedic surgeons” described by Patel et al. [23], and reduce the occurrence of so-called defensive medicine, with its vicious circle of increasing medical costs without any benefit to the patient [13, 24].

Corresponding to about 65% of all claims analyzed in the present study, lower limb surgical procedures are at a higher risk for malpractice claims than all other site surgeries [7]. In other published experiences, a tendency for malpractice claims to focus on the lower limbs was noted for the elective surgery subgroup [13], which may be a more lower-limb-focused branch than the more ubiquitous post-traumatic surgery. Claims focusing on iatrogenic complications of hip and knee replacement surgeries strongly show this trend, with sciatic nerve injury the crucial adverse event for hip replacement surgeries, and postoperative prosthesis infection the crucial adverse event for knee replacement surgeries [25]. Claims about knee replacement surgery failures often relate to the implantation of an oversized prosthesis, and it is believed that the strict use of complete preoperative checklists could dramatically limit such claims [26]. The correct use of checklists could positively impact on all orthopedic nontechnical mistakes, particularly surgery at the wrong site [2, 13, 27]. Even though surgery at the wrong site is not especially infrequent [28], there were no claims from cases of wrong-site surgery inside the studied archive.

More than 75% of the claims in the studied archive concerned alleged team malpractice, either by pure orthopedic teams (with intraoperative complication as the crucial adverse event) or by spurious multidisciplinary teams (with a missing radiographic diagnosis of bone fractures being the crucial mistake for teams made up of orthopedists and radiologists). Claims targeting purely orthopedic teams were not as frequent as seen for other published experiences [7], possibly because of an experimental selection bias relating to shifting from a study of preliminary malpractice claims to a study of final civil judgements—different members of the same team can in fact choose different strategies regarding the pretrial management of the malpractice claim.

As already found in other experiences [29], our results confirm that only rarely (9.5%) do orthopedic malpractice claims concern the clinical management of the patient, ending with the death of the patient himself. Alleged orthopedic malpractice is mainly linked to postoperative disability, but there does not seem to be a linear relationship between the severity of the disability and the statistical risk of receiving subsequent malpractice claims [9, 13].

There was technical confirmation of orthopedic malpractice for more than 50% of the malpractice claims in the archive. Once again, the corresponding value in the same archive for anesthesiologists was lower (39%, *p* < 0.05) [12]. In the present study, the final confirmation of orthopedic malpractice came after a collegial evaluation of the case involving a specialized forensic pathologist and a specialized orthopedist. A previous Italian experience concerning final civil judgements [7] had a higher percentage of claims that received final technical confirmation of orthopedic malpractice (75%, *p* < 0.05). This difference can be easily explained by the fact that cases with a stronger technical basis for alleged malpractice (and therefore with a more favorable judicial outlook) are more likely to be taken to civil court by the claimant and their forensic team (lawyers and medical experts). As far as the authors are aware, as a rough general rule, claims involving nontechnical clinical mistakes are generally resolved more quickly outside the courtroom, while technical mistakes are more likely to become a focus of cross-examination in a formal courtroom discussion.

According to our results, claims concerning nosocomial infections (14% of all claims) related mainly to elective prosthetic procedures on hips or knees. Litigation after knee replacement surgery has already been associated with an epidemic of perioperative infections [25]. It also seems that infections from post-traumatic surgery are less likely to trigger claims, possibly because such patients reason that the infection is linked to the original trauma rather than to the therapeutic surgery. Our results also seem to confirm that the majority of the claims concerning nosocomial infections are associated with technical confirmation of medical malpractice and successful litigation [15].

In our experience, the percentage of malpractice-induced DVT/PTE episodes was much lower than the percentage of malpractice-induced nosocomial infections. DVT/PTE prophylaxis is often difficult [30, 31], and the only way to reduce the risk of a malpractice claim is to choose the right prophylaxis, provide complete information to bed-restricted patients, and to keep good clinical reports on all of the preventive measures put in place. The worst outcome for DVT/PTE is obviously patient death, which is often clinically misdiagnosed. In such cases, forensic pathologists can get involved in order to establish the cause of death and the quality of the prophylaxis performed, bearing in mind that automatic equivalence between the occurrence of a PTE episode and prophylactic malpractice must be strictly avoided.

Therefore, our data analysis indicates that orthopedics is a medical specialty that has a high risk for malpractice claims. Most of the claims studied here originated via civil litigation, and malpractice was mainly suspected in perioperative and operative cases arising in general hospitals. The anatomical sites most commonly invoked in claims were the hip or the knees (40% of all claims), and sciatic nerve lesions were the main contributor. Malpractice was ascertained in about half of the analyzed claims, which were typically cases of elective surgery resulting in final permanent impairment of the patient. Conversely, death from orthopedic malpractice was rare.

Unfortunately, studies aimed at analyzing malpractice often differ considerably in the experimental methods they use, which hinders comparisons between populations and the findings of different scientific papers. As previously declared, multicenter and transnational registers should be implemented, and registers should be set up by scientific societies, since this would facilitate method sharing and permit comparisons of malpractice data from different countries, thus leading to a better understanding of this phenomenon.
